# The influence of gender stereotypical primes on the neural processing of words and faces

**DOI:** 10.1093/scan/nsaf031

**Published:** 2025-04-16

**Authors:** Luana Serafini, Francesca Pesciarelli

**Affiliations:** Department of Biomedical, Metabolic, and Neural Sciences, University of Modena and Reggio Emilia, Via Campi 287, 41100, Modena, Italy; Department of Biomedical, Metabolic, and Neural Sciences, University of Modena and Reggio Emilia, Via Campi 287, 41100, Modena, Italy

**Keywords:** gender stereotype, event-related potentials (ERPs), face processing, word processing, N400, P300

## Abstract

Implicit and automatic gender stereotyping and its neural correlates have been extensively investigated in language. This study aimed to extend this investigation to human face processing. We recorded response times (RTs) and Event-Related Potentials (ERPs) to a target third-person singular pronoun (*lui* ‘he’ or *lei* ‘she’) or face (male, female), preceded by grammatically marked or stereotypically associated words (e.g. *laureata* ‘graduated’, *badante* ‘caregiver’). Participants gender-categorized the target pronoun or face. The RTs showed a priming effect for the grammatical condition for pronouns and both grammatical and stereotypical conditions for faces. At the ERP level, feminine pronouns elicited a larger P300 and LPP (limited to men) when preceded by grammatically masculine than feminine primes. Faces elicited a larger N400, P300, and LPP (limited to women for female faces) when preceded by grammatically gender-incongruent than -congruent primes. Critically, faces showed an ERP gender stereotype asymmetry: larger N400 to male faces, and larger P300 to female faces, when preceded by stereotypically gender-incongruent than -congruent primes. This study shows that faces are influenced by gender stereotypes similarly and more strongly than linguistic stimuli. Given the multidimensionality of faces, this study is a gate-opener for future studies on the interplay between different stereotypes.

## Introduction

Imagine you were about to undergo surgery: the nurse refers to the surgeon as ‘she’ or the female surgeon’s face appears in front of you. These scenarios violate your expectations of the person’s gender based on information on their occupation. Gender stereotype is a generalized and socially shared knowledge associated with men and women, useful to simplify social interactions but harmful if used normatively (Ellemers, 2018). To implicitly probe the strength of the association between stereotypical knowledge and gender (i.e. implicit stereotyping) the priming paradigm is generally used (Meyer and Schvaneveldt, 1971; Neely, 1977). In this paradigm, two stimuli—a prime and a target—are presented sequentially, one expressing the stereotypical information and one an exemplar’s gender or vice versa. Usually, the time between prime and target onset (i.e. the stimulus onset asynchrony, SOA) is short (<350 ms) to avoid strategical/intentional processing and the target is categorized by gender (Kidder *et al.*, 2018). This paradigm generally results in faster categorization of the target when primed by stereotypically gender-congruent stimuli, suggesting that gender stereotyping can be implicitly accessed and automatically activated (Banaji and Hardin, 1996; Cacciari and Padovani, 2007; Casado *et al.*, 2023). More recently, the Event-Related Potential (ERP) technique (Luck, 2014) has accompanied the priming paradigm to probe the neural temporal dynamics of the priming effects due to gender stereotyping (see Porkert *et al*., 2024 for a review).

Most ERP studies investigated how gender stereotype violations impact brain activity in a linguistic context (Osterhout *et al.*, 1997; Lattner and Friederici, 2003; White *et al.*, 2009; Irmen *et al.*, 2010; Siyanova-Chanturia *et al.*, 2012; Canal *et al.*, 2015; Fabre *et al.*, 2015; Molinaro *et al.*, 2016; Su *et al.*, 2016; Wang *et al.*, 2016, 2017; Proverbio *et al.*, 2017, 2018; Pesciarelli *et al.*, 2019). So far, two ERP components showed a differential response to a gender-revealing word when preceded by incongruent than congruent gender stereotypes: the N400 and the P300/P600. The N400 is a negative-going deflection observed about 300–500 ms in response to targets presented within semantically incongruent contexts (Kutas and Federmeier, 2011). In priming paradigms, it showed larger amplitude to third-person singular pronouns preceded by stereotypically gender-incongruent than -congruent prime words (e.g. Babysitter—He; Babysitter—She) (Siyanova-Chanturia *et al.*, 2012; Pesciarelli *et al.*, 2019). It also showed this effect for words presented embedded in sentences (e.g. ‘Prepared the tomato sauce and then SHAVED’; ‘Fed the little girl and went to the LADY-HAIRDRESSER’) (Molinaro *et al.*, 2016; Proverbio *et al.*, 2017, 2018) or even when sentences with a gender stereotype content were spoken by a gender-incongruent than -congruent voice (Van Berkum *et al.*, 2008; Grant *et al.*, 2020). The incongruence N400 effect has also been observed in response to a stereotype-conveying word when preceded by a gender-revealing noun—i.e. the inverse situation (e.g. Men—Nurturing; Women—Nurturing) (White *et al.*, 2009; Wang *et al.*, 2016, 2017; Du and Zhang, 2023). The N400 sensitivity to stereotypical violations in language has been interpreted as a difficulty in retrieving information associated with a word from semantic memory based on the retrieval cues provided by the context (Kutas *et al.*, 2006), or the difficulty of integrating the already extracted meaning of a word with the preceding discourse (Friederici *et al.*, 2002; Hagoort, 2007).

The P300/P600/LPP refers to a family of later positive components also typically observed in linguistic studies on stereotypes. The classic P600 is a positive-going deflection appearing about 500–900 ms in case of morpho-syntactic violations, or integration difficulty that needs a formal reanalysis and repair (Niharika & Prema Rao, 2020; Osterhout and Holcomb, 1992, 1995). Its amplitude was larger when gender-revealing anaphors (e.g. reflexive pronouns) and their antecedents were embedded in sentences that were stereotypically incongruent rather than congruent (e.g. ‘the doctor prepared herself for the operation’; ‘the nurse prepared himself for the operation’) (Osterhout *et al.*, 1997; Irmen *et al.*, 2010; Canal *et al.*, 2015; Su *et al.*, 2016; Proverbio *et al.*, 2018), or when sentences with a gender stereotype content were spoken by a gender-incongruent than -congruent voice (Lattner and Friederici, 2003). The P600 sensitivity to stereotypical violations in language has been interpreted as if stereotypical gender information is encoded in the grammar, thus producing ‘syntactic’ P600 effects (Osterhout *et al.*, 1997), or as the difficulty of linking an anaphor to its antecedent (Canal *et al.*, 2015). In priming paradigms, the P300 and the Late Positive Potential (LPP), often associated with the P600, and indexing context updating (Donchin, 1981) and the salience of incongruent stimuli (Hajcak *et al.*, 2010), respectively, showed no sensitivity to gender stereotype violations (White *et al.*, 2009; Siyanova-Chanturia *et al.*, 2012; Pesciarelli *et al.*, 2019).

Compared to the investigation of gender stereotypes in verbal communication, the investigation of gender stereotypes outside of the linguistic domain is limited. In everyday life, it is often the case that we *see* the people we receive information about, and mostly we see their faces. Faces like pronouns unequivocally convey gender (Mouchetant-Rostaing *et al.*, 2000; Ito and Urland, 2003; Dickter and Bartholow, 2007) but more than pronouns or other anaphors they are ubiquitous, evolutionary relevant, and exceptionally informative in our everyday social environment. Considering that stereotypes are about people we typically see, it is surprising that only a few ERP studies used faces alongside images or words to examine the neural temporal dynamics of the interplay between gender stereotype activation and face processing (Ma *et al.*, 2008; Zhang *et al.*, 2018; Rodríguez-Gómez *et al.*, 2020; Sato *et al.*, 2020; Wu *et al.*, 2020). These studies suggested that analogous to the N400 and P300/P600 ERP components were also sensitive to gender stereotype violations elicited by faces. For instance, Zhang *et al.* (2018) found that the N400 response to a prime face, female or male, was larger when it was subsequently flanked by a stereotypically gender-incongruent than -congruent tool (e.g. spoon, hammer). Further, Sato *et al.* (2020) found that the N300, analogous to the N400 specific to picture stimuli and reflecting semantic expectancy and categorization (Barrett and Rugg, 1990; McPherson and Holcomb, 1999), was larger to faces, female or male, preceded by gender-incongruent than -congruent objects (e.g. necklace, necktie). Using faces and words, Rodríguez-Gómez *et al.* (2020) presented participants with sentences stereotypically associated with men or women (e.g. ‘His/her favourite toy as a child was a Barbie doll’) followed by male and female faces. They found that the LPP to male faces was larger when preceded by gender-incongruent than -congruent stereotypical sentences. Instead, the LPP to female faces depended on the participant’s gender: men showed a typical effect while women showed an opposite effect (i.e. larger LPP to female faces preceded by congruent than incongruent stereotypical sentences).

These studies suggest that the neural processing of gender stereotype violation may be similar when elicited by faces and words. However, they accessed gender stereotypes explicitly (i.e. the stereotypical association between prime and target was task-relevant) (Zhang *et al.*, 2018; Sato *et al.*, 2020) or did not properly avoid strategic processing (e.g. used an unconstraint or long SOA) (Rodríguez-Gómez *et al.*, 2020; Sato *et al.*, 2020). Thus, the neural correlates of the implicit and automatic processing of faces in a stereotypical context remain so far unexplored. This study aims to fill this gap by taking a novel approach to investigate gender stereotyping. We modified the word-word priming paradigm we used in Siyanova-Chanturia *et al.* (2012) and Pesciarelli *et al.* (2019) (originally adapted from Banaji and Hardin, 1996) to include both words and faces (see Roch *et al.*, 2020, for a similar manipulation). Specifically, we recorded response times (RTs) and ERPs while Italian-speaking participants performed a priming paradigm with prime words conveying a female-oriented or male-oriented gender stereotype (e.g. *insegnante* ‘teacher’, *conducente* ‘driver’), followed by either the feminine or the masculine third-person singular pronoun (i.e. *lui* ‘he’, *lei* ‘she’) (as in our previous studies), or a female or a male face, as targets. Participants gender-categorized the pronoun or the face target. As in our previous studies, a grammatical condition was included as a control condition, consisting of the same pronouns and faces being primed by words that grammatically agreed or disagreed with the target’s gender (i.e. words ending by the feminine -a or masculine -o, e.g. *inesperto*_MASC_, *antipatica*_FEM_).

We expected to replicate grammatical and stereotypical effects on language processing: faster RTs to pronouns primed by a gender-congruent than -incongruent grammatical or stereotypical word; larger ERPs (N400, P300, LPP) to gender-incongruent than -congruent targets in the grammatical condition (Osterhout *et al.*, 1997; Siyanova-Chanturia *et al.*, 2012; Canal *et al.*, 2015; Su *et al.*, 2016; Wang *et al.*, 2017; Pesciarelli *et al.*, 2019) and larger N400 to gender-incongruent than -congruent targets in the stereotypical condition (Siyanova-Chanturia *et al.*, 2012; Pesciarelli *et al.*, 2019). As a novel finding, we expected similar but greater RT and N400 ERP effects for face processing within a stereotypical context (see Zhang *et al.*, 2018 and Sato *et al.*, 2020), considering that face stimuli hold a special status in social interactions. Based on Rodríguez-Gómez *et al.* (2020), we also expected a gender stereotype violation effect for face targets on later positive components (P300/LPP), which so far showed no sensitivity to gender stereotypes in language.

## Methods

Methods are fully detailed in the ‘Supplementary methods’ section of the ‘Supplementary material’.

### Participants

Forty-two native Italian speakers participated in the experiment (21 women, age range = 18–37 yrs, *M* = 22.74 yrs, *SD* = 4.63 yrs). Eight participants were excluded from all analyses: one due to low accuracy (< 90% overall), one due to low compliance, and six due to a high number of EEG artefacts. The final sample consisted of 34 participants (17 women, two ambidextrous and one left-handed, age range = 18–37 yrs, *M* = 22.94 yrs, *SD* = 4.75 yrs). All participants had normal or corrected-to-normal vision and declared no history of neurological disorders. The final sample coincided with the sample size calculated a priori using G*Power (Faul *et al*., 2007) for the interaction effects: a sample size of 34 participants resulted adequate to detect an effect size of *η_p_*^2^= 0.06 (*f* = 0.25) with 80% power and a significance level of 0.05.

The local Ethical Committee approved the experiment. Participants provided written informed consent prior to the participation, and university students received course credits.

### Stimuli

Prime stimuli were the same used in Siyanova-Chanturia *et al.* (2012) and Pesciarelli *et al.* (2019). They consisted of 150 Italian words conveying occupation, individual characteristics, or roles. Thirty were stereotypically female and 30 stereotypically male bigender words (e.g. *insegnante* ‘teacher’, *conducente* ‘driver’, respectively); 30 were grammatically feminine and 30 grammatically masculine words with no stereotypical association (e.g. *passeggera* ‘passenger’, *pensionato* ‘pensioner’, respectively); and 30 were bigender words with no stereotypical association (e.g. *conoscente* ‘acquaintance’), these served as fillers to prevent participants from noticing the presence of the gender stereotypical words. Because the Italian language is characterized by a gender-to-ending consistency (i.e. -a ending associated with female, -o ending associated with male), we chose only bigender words ending with -e or consonant to serve as stereotypical and filler words, so that when presented in isolation they could be equally attributed to men and women. Grammatically feminine and masculine words had the gender morphologically expressed by the final vowel, -a for feminine and -o for masculine. Each word appeared either with a feminine or masculine inflection but not with both (e.g. *laureato*_MASC_ or *laureata*_FEM_, ‘graduated’).

Target stimuli consisted of either the Italian third-person singular pronouns, masculine or feminine, *lui* ‘he’ and *lei* ‘she’ [as in Siyanova-Chanturia *et al.* (2012) and Pesciarelli *et al.* (2019)], or of a female and a male face exemplar.

Each prime word was paired with the masculine or feminine third-person singular pronoun, LUI or LEI, resulting in two grammatically congruent (*inesperto*_MASC_ ‘nonexpert’—*lui, antipatica*_FEM_ ‘unpleasant’*—lei*), two grammatically incongruent (*ammalato*_MASC_ ‘sick’—*lei, eccentrica*_FEM_ ‘eccentric’*—lui*), two stereotypically congruent (*pugile* ‘boxer’*—lui*, babysitter*—lei*) and two stereotypically incongruent (*falegname* ‘carpenter’*—lei, colf* ‘maid’—*lui*) conditions.

Each prime word was also paired with the female or male face, resulting in two grammatically congruent (*inesperto*_MASC_ ‘nonexpert’—male face, *antipatica*_FEM_ ‘unpleasant’*—*female face), two grammatically incongruent (*ammalato*_MASC_ ‘sick’—female face, *eccentrica*_FEM_ ‘eccentric’*—*male face), two stereotypically congruent (*pugile* ‘boxer’*—*male face, babysitter*—*female face), and two stereotypically incongruent (*falegname* ‘carpenter’*—*female face, *colf* ‘maid’—male face) conditions. Each condition consisted of 30 trials, for a total of 480 experimental trials.

Each filler was also paired with the masculine or feminine pronouns or the female or male faces. The filler condition consisted of 120 trials. Participants completed a total of 600 trials.

### Design and procedure

**Fig. 1.**
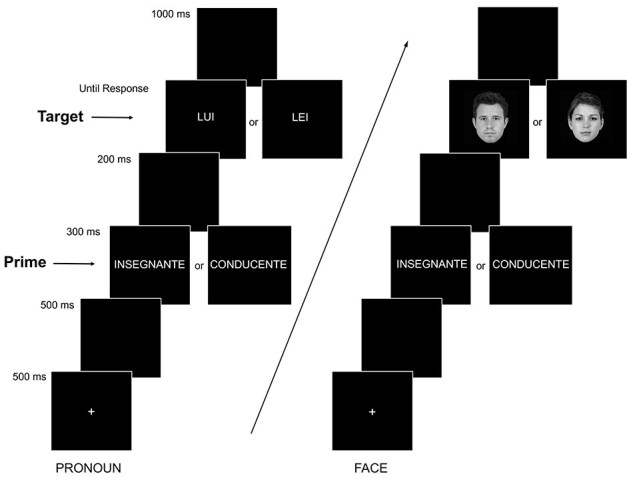
Examples of the experimental procedure used in the present study. Displayed are the stereotypically female (i.e. the prime is *insegnante* ‘teacher’) and the stereotypically male (i.e. the prime is *conducente* ‘driver’) conditions. In the pronoun condition (left panel), the target is a third-person pronoun, either gender-incongruent (*insegnante*—*lui* ‘he’; *conducente*—*lei* ‘she’) or gender-congruent (*insegnante*—*lei; conducente*—*lui*) with the prime. In the face condition (right panel) the target is a face, either gender-incongruent (*insegnante*—male face; *conducente*—female face) or gender-congruent (insegnante—female face; conducente—male face) with the prime. Displayed are the two face exemplars used in the experiment.

Participants were comfortably seated in a darkened, electrically shielded, and sound-attenuated room. An example of the experimental procedure is illustrated in Figure 1.

We used the same procedure as in Siyanova-Chanturia *et al.* (2012). In the centre of the monitor, a fixation cross (+) appeared for 500 ms (note, in Siyanova-Chanturia et al. participants pressed the spacebar to end the fixation cross and start the trial). A blank screen appeared for 500 ms, then the prime was displayed on the screen for 300 ms followed by a blank screen for 200 ms. Subsequently, the target pronoun (LUI or LEI) or the target face (female or male) appeared and remained on the screen until the participant’s response. A blank screen of 1000 ms followed each trial. Participants were instructed to decide, as quickly and as accurately as possible, whether the pronoun was grammatically masculine or feminine, or whether the face was male or female. Participants responded by pressing one of two keys, counterbalanced (left, right) across participants, using their right and left indices.

Trials were organized in six blocks, three with target pronouns and three with target faces, presented interchanged. Each block contained 100 trials: 40 with grammatical primes (20 masculine and 20 feminine), 40 with stereotypical primes (20 male-oriented and 20 female-oriented), and 20 with filler primes. Each prime was followed by all targets (*lui, lei*, male face, and female face), thus appearing four times across the whole experiment. Repetitions appeared in different blocks and the blocks’ order was counterbalanced across participants. Prime-target pairs were randomized within each block prior to presentation.

Before the experiment, participants completed a training session consisting of 20 prime-target pairs, 10 with target pronouns, and 10 with target faces. All conditions were presented once, with different stimuli than those used in the experimental session.

After the experiment, to measure individual stereotypical gender attitudes participants completed the *Bem Sex Role Inventory* (Gaudreau, 1977) and the *Ambivalence Sexism Inventory* (Glick and Fiske, 1996) (see the ‘Implicit and explicit measures of stereotyping’ section of the ‘Supplementary material’ for analyses of these questionnaires).

### EEG recording and analysis

EEG was recorded at a sampling rate of 1000 Hz with an ActiCHamp Plus (BrainProducts) system from 64 active electrodes (ActiCap Slim, BrainProducts). The EEG signal was downsampled to 500 Hz, re-referenced to the average mastoid activity, band-pass filtered from 0.01 to 80 Hz, corrected for ocular and motion artefacts, and 200 ms-baseline corrected.

Based on visual inspection of grand-averaged ERP waveforms and in line with previous literature (e.g. Siyanova-Chanturia *et al.*, 2012; Pesciarelli *et al.*, 2019), the following components were identified for target onset at frontal (F3, Fz, F4), central (C3, Cz, C4), and parietal (P3, Pz, P4) scalp sites: N400 from 200 to 350 ms after target onset; P300 from 300 to 550 ms after target onset; LPP from 650 to 850 ms after target onset. For each ERP component amplitude was measured as mean activity within the respective time window. Given the novelty of the paradigm, we complemented our *a-priori* analysis with a data-driven approach to investigate effects not constrained by predefined electrodes and time windows. Individual averages per electrode per condition were imported in Brainstorm (Tadel *et al.*, 2011), where the signal was downsampled to 125 Hz and low-pass filtered at 30 Hz, to reduce multiple testing and increase the signal-to-noise ratio (see Luck, 2014).

### Statistical analyses

Behavioural analyses were conducted only on trials with response latencies within ± 2SD from each mean. To investigate priming effects (i.e. a target gender by prime gender interaction) as a function of the type of prime and type of target, the mean RTs of correct responses per condition and the mean ERP amplitudes of correct trials were submitted to repeated-measure ANOVAs with the within-subject factors: Prime Gender (female, male), Target Gender (female, male), Prime Type (grammatical, stereotypical), and Target Type (pronoun, face). Longitude (anterior, central, posterior) and Latitude (left, midline, right) were additional within-subject factors only for ERP analyses. The levels corresponded to the mean activity of F3, Fz, F4 (Anterior), C3, Cz, C4 (Central), P3, Pz, P4 (Posterior), F3, C3, P3 (Left), Fz, Cz, Pz (Midline), and F4, C4, P4 (Right). Hit rates were not analysed due to the ceiling effect, with all conditions averaging 96%–98% correct.

We additionally performed the same ANOVAs including the between-subject factor Participant Gender (woman, men) and separate ANOVAs on pronoun and face targets. Degrees of freedom were adjusted according to the Greenhouse–Geisser method; only corrected significance levels are reported. The level of significance testing was *P* = .05. Planned contrasts were performed to further investigate the priming effect across all combinations of Prime Type and Target Type. Main effects and interactions not involving a priming effect are not focal to the question under study and thus are not reported.

To investigate effects unconstrained by *a-priori* assumptions, we performed a mass-univariate analysis. Specifically, we conducted point-by-point two-tailed paired Student’s *t*-tests with time points of 8 ms, in the 200–850 ms poststimulus time window, wherein the effects of interest are predicted to occur, comparing the incongruent to the congruent condition for all conditions at all electrode sites (excluded TP9, TP10, FT10, FT9, and the outermost ring of electrodes, which are of no interest). The level of significant testing was *P *= .05, corrected for multiple testing using the False Discovery Rate (FDR) (Benjamini and Hochberg, 1995).

## Results

### Behavioural results

The omnibus ANOVA conducted on the mean correct RTs yielded a statistically significant Prime Gender × Target Gender interaction (*F*(1,33) = 37.30, *P* < .001, *η_p_*^2^= 0.53, 90% CI [0.32, 0.65]), a Prime Gender × Target Gender × Target Type interaction (*F*(1,33) = 12.24, *P* = 0.001, *η_p_*^2^= 0.27 [0.08, 0.44]), a Prime Gender × Target Gender × Prime Type interaction (*F*(1,33) = 17.45, *P* < .001, *η_p_*^2^= 0.35 [0.13, 0.51]), and a Prime Gender × Target Gender × Prime Type × Target Type interaction (*F*(1,33) = 6.73, *P* = .01, *η_p_*^2^= 0.17 [0.02, 0.35]).

Separate ANOVAs for the pronoun and face targets revealed a statistically significant Prime Gender × Target Gender interaction [for the pronoun: *F*(1,33) = 10.17, *P* = .003, *η_p_*^2^= 0.24, [0.05, 0.41]; for the face: *F*(1,33) = 56.48, *P* < .001, *η_p_*^2^= 0.63 [0.44, 0.73], and a Prime Gender × Target Gender × Prime Type interaction [for the pronoun: *F*(1,33) = 4.40, *P* = .04, *η_p_*^2^= 0.12 [0, 0.29]; for the face: *F*(1,33) = 29.12, *P* < .001, *η_p_*^2^= 0.47 [0.25, 0.61].

**Fig. 2.**
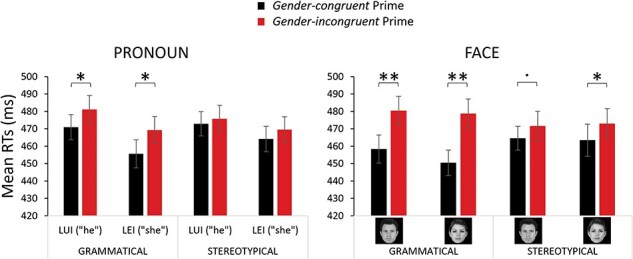
Mean correct RTs to discriminate the gender of the target pronoun (left panel) or target face (right panel) when primed by a grammatically or stereotypically gender-congruent or -incongruent word.

RTs were faster when pronouns were preceded by congruent than incongruent grammatical primes, but not when they were preceded by congruent than incongruent stereotypical primes. Similarly, RTs were faster when faces were preceded by congruent than incongruent grammatical primes, but also when the female face was preceded by congruent than incongruent stereotypical primes. The male face preceded by stereotypical primes showed a tendency for a gender congruency effect not to reach a significance level (see Table 1 and Figure 2).

**Table 1.** Descriptive statistics and planned pairwise contrasts (one-tailed T-tests: incongruent > congruent) for correct RTs in milliseconds to gender-categorize the target pronouns and faces as a function of the prime type (grammatical, stereotypical) and gender (congruent, incongruent).

Target	Prime	*M(SD)*	*d*[95% CI]	*df*	*t*	*p*_(FDR)_
**Pronoun**	**Grammatical**					
Masculine	Congruent	470.95(42.44)	−0.42[−∞, −0.12]	33	−2.44	0.02*
	Incongruent	481.12(46.51)				
Feminine	Congruent	455.61(47.17)	−0.48[−∞, −0.17]	33	−2.78	0.01*
	Incongruent	469.28(45.47)				
	**Stereotypical**					
Masculine	Congruent	472.90(40.80)	−0.12[−∞, 0.17]	33	−0.68	0.25
	Incongruent	475.83(44.83)				
Feminine	Congruent	464.16(42.47)	−0.23[−∞, 0.06]	33	−1.34	0.11
	Incongruent	469.51(43.55)				
**Face**	**Grammatical**					
Male	Congruent	458.40(47.37)	−0.77[−∞, −0.44]	33	−4.46	0.004**
	Incongruent	480.45(48.07)				
Female	Congruent	450.51(42.66)	−1.42[−∞, −1.01]	33	−8.29	0.004**
	Incongruent	478.76(48.77)				
	**Stereotypical**					
Male	Congruent	464.62(40.06)	−0.31[−∞, −0.02]	33	−1.82	0.052
	Incongruent	471.63(49.21)				
Female	Congruent	450.51(42.66)	−0.43[−∞, −0.13]	33	−2.51	0.02*
	Incongruent	478.76(48.77)				

* *P* < .05, **P < .01.

Participant’s Gender showed no significant impact on any of the effects of interest (all *P*s >0.10).

### ERP results

#### N400

The omnibus ANOVA showed a statistically significant Prime Gender × Target Gender interaction (*F*(1,33) = 8.92, *P* = .005, *η_p_*^2^= 0.21, 90% CI = 0.04, 0.39), as well as a statistically significant Prime Gender × Target Gender × Longitude interaction (*F*(1.40,46.26) = 5.07, *P* = .02, *η_p_*^2^= 0.13 [0.01, 0.28]). It also showed tendencies for a Prime Gender × Target Gender × Prime Type interaction (*F*(1,33) = 4.01, *P* = .054, *η_p_*^2^= 0.11 [0, 0.28]), and for a Prime Gender × Target Gender × Prime Type × Target Type interaction (*F*(1,33) = 3.20, *P* = .08, *η_p_*^2^= 0.09 [0, 0.25]) which did not reach significance level.

**Fig. 3.**
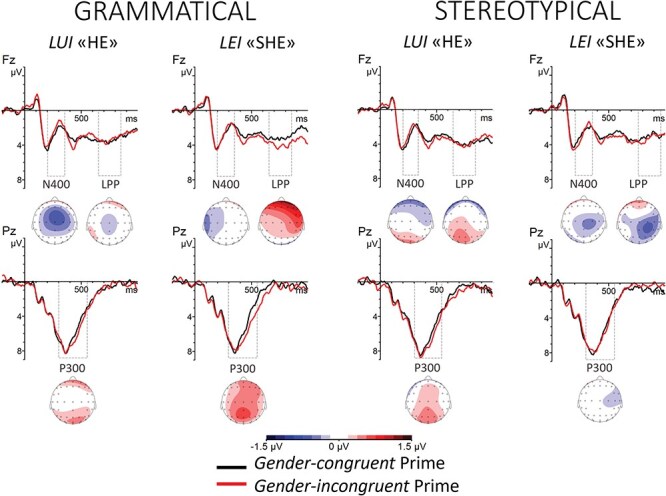
Grand-averaged ERP waveforms in response to the target pronoun LUI (‘he’) (left plots) or LEI (‘she’) (right plots) when primed by a grammatically (left panels) or stereotypically (right panels) gender-congruent (in black or dark grey) or -incongruent word (in red or light grey). Plotted are electrodes Fz, representative of the N400 and LPP effects, and Pz, representative of the P300 effects. ERPs are time-locked to the target pronoun onset. Negative voltages are plotted upward. Below each graph, topographical scalp maps represent the distribution of the mean incongruent-minus-congruent effect for each time window and condition.

The ANOVA for the pronoun target only revealed a tendency for a Prime Gender × Target Gender × Prime Type × Longitude × Latitude interaction, which did not reach a significance level (*F*(2,27.75) = 2.38, *P* = .06, *η_p_*^2^= 0.07 [0, 0.31]). Specifically, the N400 tended to be more negative for the masculine pronoun preceded by incongruent than congruent grammatical primes at the central electrode cluster (see Table 2, Fig. 3).

**Table 2.** Descriptive statistics and planned pairwise contrasts (one-tailed T-tests: incongruent < congruent) for the mean N400 amplitude (µV) to the target pronouns and faces as a function of the prime type (grammatical, stereotypical) and gender (congruent, incongruent) at anterior (F3, Fz, F4) and central (C3, Cz, C4) electrode clusters.

Target	Prime	Electrode Cluster	*M(SD)*	*d*[95% CI]	*df*	*t*	*p*_(FDR)_
**Pronoun**	**Grammatical**	**Anterior**					
Masculine	Congruent		2.40(2.03)	0.11[−0.18, ∞]	33	0.63	0.39
	Incongruent		2.24(2.08)				
Feminine	Congruent		2.34(1.76)	0.004[−0.28, ∞]	33	0.02	0.61
	Incongruent		2.33(2.09)				
		**Central**					
Masculine	Congruent		4.12(2.20)	0.30[0.01, ∞]	33	1.78	0.096
	Incongruent		3.70(2.36)				
Feminine	Congruent		4.14(2.39)	0.17[−0.12, ∞]	33	0.98	0.27
	Incongruent		3.92(2.52)				
	**Stereotypical**	**Anterior**					
Masculine	Congruent		2.94(2.03)	0.20[−0.09, ∞]	33	1.16	0.23
	Incongruent		2.61(1.80)				
Feminine	Congruent		2.56(2.19)	−0.03[−0.31, ∞]	33	−0.17	0.65
	Incongruent		2.61(2.67)				
		**Central**					
Masculine	Congruent		4.15(2.37)	0.08[−0.21, ∞]	33	0.45	0.44
	Incongruent		4.04(2.39)				
Feminine	Congruent		4.19(2.29)	0.24[−0.05, ∞]	33	1.39	0.17
	Incongruent		3.86(2.82)				
**Face**	**Grammatical**	**Anterior**					
Male	Congruent		−1.11(2.37)	0.69[0.37, ∞]	33	4.05	0.02*
	Incongruent		−2.15(2.01)				
Female	Congruent		1.22(2.64)	0.31[0.02, ∞]	33	1.82	0.096
	Incongruent		−1.69(2.23)				
		**Central**					
Male	Congruent		1.58(2.08)	0.44[0.14, ∞]	33	2.58	0.03*
	Incongruent		0.93(1.94)				
Female	Congruent		1.63(2.37)	0.44[0.14, ∞]	33	2.56	0.03*
	Incongruent		1.08(2.18)				
	**Stereotypical**	**Anterior**					
Male	Congruent		−0.81(2.49)	0.48[0.17, ∞]	33	2.78	0.03*
	Incongruent		−1.44(2.32)				
Female	Congruent		−1.50(2.28)	−0.27[−0.56, ∞]	33	−1.60	0.94
	Incongruent		−1.08(2.13)				
		**Central**					
Male	Congruent		1.91(2.15)	0.46[0.16, ∞]	33	2.69	0.03*
	Incongruent		1.40(2.01)				
Female	Congruent		1.22(2.24)	−0.28[−0.57, ∞]	33	−1.64	0.94
	Incongruent		1.54(2.16)				

* *P* < .05.

**Fig. 4.**
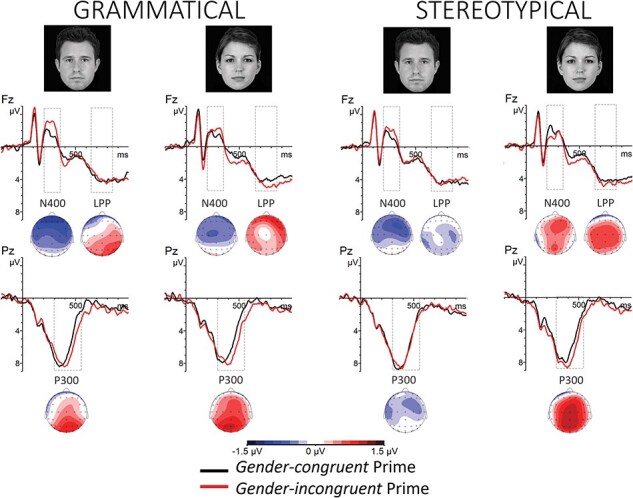
Grand-averaged ERP waveforms in response to the MALE (left plots) or FEMALE (right plots) target face when primed by a grammatically (left panels) or stereotypically (right panels) gender-congruent (in black or dark grey) or -incongruent word (in red or light grey). Plotted are electrodes Fz, representative of the N400 and LPP effects, and Pz, representative of the P300 effects. ERPs are time-locked to the target face onset. Negative voltages are plotted upward. Below each graph, topographical scalp maps represent the distribution of the mean incongruent-minus-congruent effect for each time window and condition.

The ANOVA for the face target revealed a statistically significant Prime Gender × Target Gender interaction (*F*(1,33) = 6.98, *P *= .01, *η_p_*^2^= 0.17 [0.02, 0.35]), a Prime Gender × Target Gender × Longitude interaction (*F*(1.43,47.30) = 5.39, *P* = .015, *η_p_*^2^= 0.14 [0.02, 0.28]) and a Prime Gender × Target Gender × Prime Type interaction (*F*(1,33) = 9.39, *P* = .004, *η_p_*^2^= 0.22 [0.05, 0.4]). The N400 was more negative in response to the male face when preceded by incongruent than congruent grammatical primes both at anterior and central electrode clusters. The N400 was also more negative in response to the female face when preceded by incongruent than congruent grammatical primes at the central electrode cluster and tendentially at the anterior electrode cluster. Corresponding effects were found for the male face preceded by incongruent than congruent stereotypical primes at anterior and central clusters. No other effect reached significance at any other cluster tested (all *P*s > .10) (see Table 2, Fig. 4).

Participant’s Gender showed no significant impact on any of the effects of interest (all *P*s > .10).

#### P300

The omnibus ANOVA yielded a statistically significant Prime Gender × Target Gender interaction (*F*(1,33) = 6.90, *P *= .01, *η_p_*^2^= 0.17, 90% CI = 0.02, 0.35), Prime Gender × Target Gender × Longitude interaction (*F*(1.47,48.43) = 4.11, *P* = .03, *η_p_*^2^= 0.11 [0, 0.25]), and Prime Gender × Target Gender × Latitude interaction (*F*(1.54,50.77) = 4.005, *P* = .03, *η_p_*^2^= 0.11 [0, 0.24]). A tendency emerged for a Prime Gender × Target Gender × Target Type × Longitude interaction which did not reach a significance level (*F*(1.51,49.97) = 2.99, *P *= .07, *η_p_*^2^= 0.08 [0, 0.21]).

The ANOVA for the pronoun target revealed a statistically significant Prime Gender × Target Gender × Prime Type × Longitude × Latitude interaction (*F*(2.92,26.82) = 3.59, *P* = .01, *η_p_*^2^= 0.10 [0.02, 0.43]). However, comparisons at the central or posterior electrode clusters did not show differences in the P300 amplitude in response to the pronoun depending on the gender congruency of the prime for grammatical or stereotypical primes (all *P*s > .10) (see Table 3, Fig. 3).

**Table 3.** Descriptive statistics and planned pairwise contrasts (one-tailed T-tests: incongruent > congruent) for the mean P300 amplitude (µV) to the target pronouns and faces as a function of the prime type (grammatical, stereotypical) and gender (congruent, incongruent) at central (C3, Cz, C4) and posterior (P3, Pz, P4) electrode clusters.

Target	Prime	Electrode Cluster	*M(SD)*	*d*[95% CI]	*df*	*t*	*p*_(FDR)_
**Pronoun**	**Grammatical**	**Central**					
Masculine	Congruent		5.75(2.29)	−0.03[−∞, 0.25]	33	−0.17	0.53
	Incongruent		5.79(2.46)				
Feminine	Congruent		5.51(2.21)	−0.26[−∞, 0.03]	33	−1.53	0.15
	Incongruent		5.83(2.52)				
		**Posterior**					
Masculine	Congruent		4.94(2.65)	−0.19[-∞, 0.10]	33	−1.08	0.29
	Incongruent		5.26(2.90)				
Feminine	Congruent		4.63(2.52)	−0.32[−∞, −0.03]	33	−1.90	0.11
	Incongruent		5.11(2.59)				
	**Stereotypical**	**Central**					
Masculine	Congruent		5.83(2.09)	−0.09[−∞, 0.19]	33	−0.50	0.45
	Incongruent		5.98(2.57)				
Feminine	Congruent		5.80(2.18)	0.13[−∞, 0.42]	33	0.78	0.83
	Incongruent		5.61(2.40)				
		**Posterior**					
Masculine	Congruent		5.18(2.74)	−0.16[−∞, 0.12]	33	−0.95	0.31
	Incongruent		5.48(2.94)				
Feminine	Congruent		4.98(2.72)	0.11[−∞, 0.25]	33	−0.20	0.53
	Incongruent		5.04(2.90)				
**Face**	**Grammatical**	**Central**					
Male	Congruent		−1.11(2.37)	−0.10[-∞, 0.19]	33	−0.57	0.45
	Incongruent		−2.15(2.01)				
Female	Congruent		1.22(2.64)	−0.27[-∞, 0.02]	33	−1.59	0.15
	Incongruent		−1.69(2.23)				
		**Posterior**					
Male	Congruent		5.58(2.62)	−0.48[-∞, −0.18]	33	−2.80	0.02*
	Incongruent		6.24(3.03)				
Female	Congruent		5.37(2.80)	−0.50[-∞, −0.20]	33	−2.92	0.02*
	Incongruent		6.27(2.77)				
	**Stereotypical**	**Central**					
Male	Congruent		4.81(1.86)	0.27[-∞,0.56]	33	1.58	0.94
	Incongruent		4.45(1.93)				
Female	Congruent		4.07(1.86)	−0.50[−∞, −0.19]	33	−2.90	0.02*
	Incongruent		4.77(2.23)				
		**Posterior**					
Male	Congruent		6.15(2.76)	0.06[-∞,0.34]	33	0.36	0.73
	Incongruent		6.05(2.77)				
Female	Congruent		5.59(2.41)	−0.56[−∞, −0.26]	33	−3.29	0.02*
	Incongruent		6.42(2.80)				

* *P* < .05.

The ANOVA for the face target revealed a statistically significant Prime Gender × Target Gender interaction (*F*(1,33) = 5.87, *P* = .02, *η_p_*^2^= 0.15 [0.01, 0.33]), a Prime Gender × Target Gender × Longitude interaction (*F*(1.63,53.78) = 6.95, *P *= .004, *η_p_*^2^= 0.17 [0.04, 0.31]), and a Prime Gender × Target Gender × Latitude interaction (*F*(1.85,60.94) = 4.35, *P* = 0.02, *η_p_*^2^= 0.12 [0.01, 0.23]). The P300 was more positive in response to the face when preceded by incongruent than congruent grammatical primes at the posterior electrode cluster. Further, the P300 was more positive in response to the female face when preceded by incongruent than congruent stereotypical primes at posterior and central electrode clusters (see Table 3, Fig. 4).

Participant’s Gender showed no significant impact on any of the effects of interest (all *P*s > .10).

#### LPP

The omnibus ANOVA yielded a statistically significant Prime Gender × Target Gender interaction (*F*(1,33) = 4.69, *P *= .04, *η_p_*^2^= 0.12, 90% CI = 0, 0.3), and a Prime Gender × Target Gender × Prime Type × Latitude interaction (*F*(1.99,65.65) = 5.40, *P* = .007, *η_p_*^2^= 0.14 [0.02, 0.26]). Since the target type, pronoun or face, showed no impact on the priming effect, we did not perform separate ANOVAs for the pronoun and face target. The LPP was more positive in response to the feminine pronoun and to the female face when preceded by incongruent than congruent grammatical primes at the anterior electrode cluster. The LPP also tended to be more positive in response to the female face when preceded by incongruent than congruent stereotypical primes at the central electrode cluster, but the effect did not reach a significance level (see Table 4, Figs 3 and 4).

**Table 4.** Descriptive statistics and planned pairwise contrasts (one-tailed T-tests: incongruent > congruent) for the mean LPP amplitude (µV) to the target pronouns and faces as a function of the prime type (grammatical, stereotypical) and gender (congruent, incongruent) at anterior (F3, Fz, F4) and central (C3, Cz, C4) electrode clusters.

Target	Prime	Electrode Cluster	*M(SD)*	*d*[95% CI]	*df*	*t*	*p*_(FDR)_
**Pronoun**	**Grammatical**	**Anterior**					
Masculine	Congruent		2.70(2.28)	−0.07[−∞, 0.21]	33	−0.43	0.63
	Incongruent		2.83(2.54)				
Feminine	Congruent		2.39(2.82)	−0.60[−∞, −0.29]	33	−3.52	0.02*
	Incongruent		3.29(2.61)				
		**Central**					
Masculine	Congruent		3.28(2.18)	0.07[−∞, 0.35]	33	0.40	0.82
	Incongruent		3.18(2.21)				
Feminine	Congruent		2.97(2.28)	−0.27[−∞, −0.02]	33	−1.58	0.25
	Incongruent		3.36(2.23)				
	**Stereotypical**	**Anterior**					
Masculine	Congruent		3.45(2.33)	0.07[−∞, 0.36]	33	0.44	0.82
	Incongruent		3.32(2.27)				
Feminine	Congruent		2.80(2.52)	−0.06[−∞, 0.22]	33	−0.34	0.63
	Incongruent		2.91(2.68)				
		**Central**					
Masculine	Congruent		3.39(2.25)	−0.07[−∞, 0.21]	33	−0.43	0.63
	Incongruent		3.52(2.42)				
Feminine	Congruent		3.18(2.23)	0.20[−∞, 0.48]	33	1.17	0.87
	Incongruent		2.85(2.43)				
**Face**	**Grammatical**	**Anterior**					
Male	Congruent		3.40(2.59)	0.15[−∞, 0.43]	33	0.88	0.87
	Incongruent		3.15(2.55)				
Female	Congruent		3.14(2.45)	−0.53[−∞, −0.22]	33	−3.08	0.02*
	Incongruent		3.95(2.35)				
		**Central**					
Male	Congruent		3.59(1.94)	−0.16[−∞, 0.12]	33	−0.94	0.47
	Incongruent		3.84(2.07)				
Female	Congruent		3.47(2.40)	−0.22[−∞, 0.06]	33	−1.30	0.33
	Incongruent		3.82(2.11)				
	**Stereotypical**	**Anterior**					
Male	Congruent		3.56(2.24)	0.002[−∞, 0.28]	33	0.01	0.73
	Incongruent		3.55(2.40)				
Female	Congruent		3.55(2.24)	−0.05[−∞, 0.24]	33	−0.27	0.63
	Incongruent		3.64(3.12)				
		**Central**					
Male	Congruent		4.02(2.08)	0.17[−∞, 0.45]	33	1.00	0.87
	Incongruent		3.71(2.18)				
Female	Congruent		3.20(1.94)	−0.41[−∞, −0.11]	33	−2.39	0.06
	Incongruent		3.80(2.67)				

* *P* < .05.

The omnibus ANOVA including the factor of Participant’s Gender revealed a statistically significant Prime Gender × Target Gender × Longitude × Participant’s Gender interaction (*F*(1.25,39.88) = 5.04, *P* = .02, *η_p_*^2^= 0.14 [0.01, 0.29]), and a tendency for a Prime Gender × Target Gender × Prime Type × Longitude × Participant’s Gender interaction (*F*(1.57,50.36) = 2.59, *P* = .097, *η_p_*^2^= 0.07 [0, 0.19]). The LPP of male participants was more positive in response to the feminine pronoun when preceded by incongruent than congruent grammatical primes at the anterior electrode cluster [*M* = 2.69, *SD* = 2.59; *M* = 1.53, *SD* = 2.91, respectively; *t*(16) = −3.24, *P* = .048, *d* = −0.79, 95% CI =—∞, −0.32]; and the LPP of female participants was more positive in response to the female face when preceded by incongruent than congruent grammatical primes at the anterior electrode cluster [*M* = 4.56, *SD* = 2.27; *M* = 3.22, *SD* = 2.27, respectively; *t*(16) =  −3.86, *P* = .03, *d* = −0.94 [−∞, −0.44].

#### Mass-univariate analysis

**Fig. 5.**
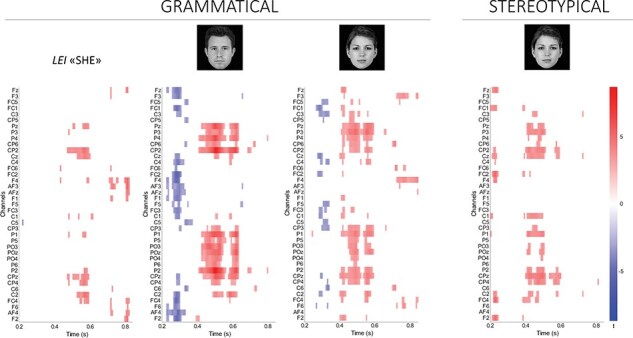
Raster plots representing the significant *t*-values with control for FDR of the priming (incongruent-minus-congruent) effect at all electrodes and time point analysed for (i) the feminine target pronoun *lei* (‘she’) in the grammatical condition (left-most panel), (ii) the male target face in the grammatical condition (central-left panel), (iii) the female target face in the grammatical condition (central-right panel), and (iv) the female target face in the stereotypical condition (right-most panel). Time from 200 to 850 ms post-target onset is shown on the x-axis and the 39 electrodes of interest on the y-axis. Results are plotted in 8 ms lags. Red (blue) indicates that ERPs in the incongruent condition are more positive (negative) than ERPs in the congruent condition.

Point-by-point paired Student’s *t*-tests comparing the incongruent to the congruent condition for the pronoun target revealed statistically significant effects only for the feminine target pronoun in the grammatical condition (*t*(33) range = 5, −3.55; *Ps*FDR < 0.002). These effects were observed from 472 to 616 ms post-target at midline and right centro-parietal electrodes sites, and from 720 to 760 ms and 800 to 824 ms post-target at fronto-central sites, showing more positive amplitude for the incongruent than congruent condition (see Fig. 5).

For the face target in the grammatical condition, point-by-point *t*-tests showed statistically significant differences in the ERP amplitudes for both female and male target faces. For the male target face, significant differences (*t*(33) range = 8.72, −5.27, *Ps*FDR < 0.008) emerged at fronto-central electrode sites, centred on the midline from 224 to 328 ms post-target onset, while more laterally distributed from 328 to 368 ms post-target onset. The ERP amplitude was more negative for the incongruent than congruent condition. Later, from 392 to 632 ms post-target onset, a statistically significant difference emerged at centro-parietal and parieto-occipital electrode sites, with ERP amplitude being more positive for the incongruent than congruent condition. Last, significant differences emerged at a few time-points and at centro-parietal and frontal electrode sites (see Fig. 5). For the female target face in the grammatical condition, significant differences (*t*(33) range = 5.02, −4.22, *Ps*FDR < 0.006) emerged from 264 to 336 ms post-target onset at frontal, central, and centro-parietal electrodes, showing more negative amplitude for the incongruent than congruent condition. Later, from 400 to 592 ms after target onset, the ERP amplitude significantly differed, being more positive to incongruent than congruent conditions at central, parietal, and parieto-occipital scalp sites. A later anterior right lateralized effect emerged from 656 to 848 ms post-target, being more positive to the incongruent than congruent condition (see Fig. 5).

For the face target in the stereotypical condition, significant results emerged only for the female target face, whereas ERP effects for the male target face resulted in nonsignificant after FDR correction. For female target faces in the stereotypical condition, ERP amplitudes significantly differed (*t*(33) range = 5.19, −2.83, *Ps*FDR < 0.004) from 200 to 240 ms after target onset at midline and right fronto-central and central electrodes, showing more negative amplitude for the *congruent* than incongruent condition. Later, from 384 to 600 ms after target onset, the ERP amplitude significantly differed at centro-parietal and parieto-occipital electrodes, showing more positive amplitude for the incongruent than congruent condition. A later shorter-lived significant difference emerged at 728 ms after target onset at C3, Cz, and C2 (see Fig. 5).

## Discussion

This study investigated the neural correlates of the implicit and automatic processing of faces in a stereotypical context by using a novel word-face priming paradigm (adapted from the word-word priming paradigm used in Siyanova-Chanturia *et al.*, 2012; Pesciarelli *et al.*, 2019) and the ERP technique. Specifically, we recorded RTs and ERPs while participants categorized by gender feminine and masculine Italian third-person singular pronouns (i.e. *lui* ‘he’, *lei* ‘she’), and female and male faces, primed by stereotypically associated or grammatically marked female or male words.

### Priming effects for target faces

At the *behavioural* level, we found priming effects for faces in both grammatical and stereotypical conditions. In the stereotypical condition, the effect was significant only for female faces, but a tendency appeared also for male faces. Our findings with faces align with existing evidence using the priming paradigm with linguistic stimuli (e.g. Banaji and Hardin, 1996; Cacciari and Padovani, 2007; Siyanova-Chanturia *et al.*, 2012; Pesciarelli *et al.*, 2019; Casado *et al.*, 2023). These effects also corroborate evidence from behavioural studies using faces in gender stereotypical contexts (Le Gal and Bruce, 2002; Becker *et al.*, 2007; Dzhelyova *et al.*, 2012; Zhu *et al.*, 2020; Fitousi, 2021), specifically those showing faster processing of gender stereotypical words following gender-congruent face primes (White *et al.*, 2018; Zhang *et al.*, 2019), further suggesting that face processing is implicitly and automatically affected by gender stereotypes.

At the ERP level, the priming effects for faces in the *grammatical* condition resembled our previous findings with pronouns (Siyanova-Chanturia *et al.*, 2012; Pesciarelli *et al.*, 2019). Specifically, the N400 and P300 were larger when faces were preceded by grammatically incongruent than congruent primes. Despite the use of different stimuli (i.e. faces compared with three-letter pronouns), the N400 and P300 latencies appeared comparable to those observed in our previous studies. Data-driven analyses more precisely determined the spatio-temporal extent of the effects, with the N400 starting as early as 216 ms and lasting until 400 ms post-target onset over fronto-central scalp areas, and the P300 starting at 400 ms and lasting until maximum 648 ms post-target onset over centro-parietal and parieto-occipital scalp areas. The N400 effect appeared more frontally distributed compared to the typical N400 effect. Similarly, in our previous studies, the effect was also observed at frontal sites (Siyanova-Chanturia *et al.*, 2012; Pesciarelli *et al.*, 2019) and the scalp distribution of the N400 is known to vary depending on the type of eliciting item (Kutas and Federmeier, 2011). For the P300 effect, we did not find the modulation by participants’ gender (Siyanova-Chanturia *et al.*, 2012; Pesciarelli *et al.*, 2019) nor the grammatical gender asymmetry (Pesciarelli *et al.*, 2019) that we observed with pronouns. This may be because the effects of grammatical violations are stronger when elicited by faces than words, thus appearing no matter the observer’s or target’s gender. A later positivity, interpreted as the LPP, instead showed a priming effect only in women as did the P300 (Pesciarelli *et al.*, 2019; Siyanova-Chanturia *et al.*, 2012; or P600: Osterhout *et al.*, 1997), which we previously interpreted as greater sensitivity of females to agreement violations supported by their greater grammatical awareness or competence (Rosenberg and Sutton-Smith, 1969). Thus, with faces, the effect could be broader and specific to women only at this later stage. However, this LPP effect was limited to female faces, different from our previous P300 effect, which was limited to masculine pronouns (Pesciarelli *et al.*, 2019). This could be interpreted considering that faces are more salient stimuli than pronouns. Specifically, own-gender faces could be more salient than other-gender faces, so women may be more sensitive to a female face (own gender) violating grammatical male agreement than to a male face (other gender) violating grammatical female agreement. Here, the LPP was frontally distributed, in line with evidence of late positivities in the frontal brain electrode sites for unexpected words in strongly constraining contexts (see Federmeier *et al.*, 2007). From these effects, it seems that face processing resembles word processing in grammatical gender violation contexts. However, the effects of grammatical violations appeared greater (not limited to an observer’s gender nor target gender, for the P300) and broader (extending to the later LPP) for faces than words.

The ERP effects of *stereotype* violation on face processing were similar but reduced as compared to the ERP effects of grammatical violation, as they previously occurred with pronouns (Siyanova-Chanturia *et al.*, 2012; Pesciarelli *et al.*, 2019). But, with pronouns, priming effects only emerged on the N400, whereas with faces they emerged on both N400 and P300. Specifically, they showed a gender asymmetry: the effect was on the N400 for violations of the female stereotype by male faces (e.g. babysitter*—*male face), whereas on the P300 for violations of the male stereotype by female faces (e.g. falegname ‘carpenter’*—*female face). Note that the N400 effect emerged in the *a-priori* analyses, but did not reach statistical significance when FDR correction was applied for multiple comparisons in the data-driven analyses. Our result echoes previous evidence of gender stereotype asymmetry in language, i.e. N400 incongruence effect only to the violation of the female-related stereotype by male agents (Proverbio *et al.*, 2018 in men; Siyanova-Chanturia *et al.*, 2012) and P600 incongruence effect only to the violation of the male-related stereotype by female agents (Irmen *et al.*, 2010; Proverbio *et al.*, 2018 in men; Su *et al.*, 2016). A stereotype asymmetry also appeared in the only study investigating the ERP correlates of face processing in a gender stereotype context using both linguistic and face stimuli (Rodríguez-Gómez *et al.*, 2020). Men showed larger LPP to both female and male faces preceded by stereotypically incongruent than congruent sentences; women showed this effect for male faces but an opposite one (i.e. larger LPP to congruent than incongruent) for female faces. The authors interpreted the opposite effect as indicating that women struggle to process compliance with a typical female stereotype because they are more aware than men of their gender stereotype. Echoing their finding, an early inverse priming effect, i.e. larger amplitude to congruent than incongruent prime-target pairs, emerged in our data-driven analyses for the female faces in the stereotypical condition. This effect from 200 to 240 ms post-target onset could correspond to an N200 effect, suggesting that the *a-priori* identified N400 time window could instead reflect an N200-N400 complex, at least for face targets. The N200 is an ERP component peaking between 180 and 300 ms poststimulus onset at fronto-central sites, indexing the selection of attention based on stimulus properties (Eimer, 1997; Kenemans *et al.*, 1993; Näätänen and Gaillard, 1983; Wijers *et al.*, 1989). This component previously showed a larger amplitude to repeated or semantically congruent stimuli (e.g. Pickering and Schweinberger, 2003; Du *et al.*, 2014; Pesciarelli *et al.*, 2021), and larger amplitude to female than male faces (e.g. Ito and Urland, 2003, 2005). Our result is consistent with the literature and might reflect more in-depth processing of female faces matching female-related stereotypes. However, since this effect was unpredicted it needs further investigation. These findings overall argue for a more complex processing of female than male stereotypes, but methodological differences between this study and ours could explain the different effects. We argue that Rodríguez-Gómez et al.’s use of stereotypical sentences, the indefinite time to process the sentences, the indefinite SOA, and the long face presentation (1000 ms) could all have allowed strategic/deliberate processes to guide the stereotype effects on face processing. On the other hand, the use of stereotypical words, the short duration of word presentation (300 ms), the short SOA (500 ms), and the short face presentation could have allowed us to detect earlier more automatic/nonstrategic effects. These effects could reflect semantic more than motivational, affective, or attentive processes.

The asymmetry possibly stems from two different processes. First, the Italian language is gender-marked, but in Italian the male deflection is commonly used in a gender-unmarked way to refer to both female and male gender (e.g. *uomo* ‘man’, referring to both a man and woman). Second, the rate with which society has evolved regarding female and male stereotypes is different: women seem to have entered male roles more than men have entered female roles (see Cacciari and Padovani, 2007). The sensitivity of the N400 to violations of the female stereotype by a male face could suggest that this scenario is processed as a semantic violation (Kutas and Hillyard, 1980; Kutas and Federmeier, 2011). Female-related stereotypical primes might have opened the ‘female’ semantic node exclusively, so that a following male target is more difficult to retrieve from semantic memory (Kutas *et al.*, 2006), or its meaning is more difficult to integrate with the preceding context (Friederici *et al.*, 2002; Hagoort, 2007). On the other hand, the sensitivity of the P300 to violations of the male stereotype by a female face could suggest that this scenario is perceived as less probable than the stereotypical one (Duncan-Johnson and Donchin, 1977; Donchin, 1981; Sutton and Ruchkin, 1984) but not as semantically disruptive as the violation of the female stereotype by a man. Male-related stereotypical primes might have opened both the ‘male’ and ‘female’ nodes in semantics, but the opening of the female node could still be perceived as less probable. In line with our interpretation, Cacciari and Padovani (2007) previously found that the activation of a male stereotype speeded up the processing of a male pronoun but did not inhibit the processing of a female pronoun, whereas the activation of a female stereotype also inhibited the processing of a male pronoun. Of note, from our paradigm it is not possible to disambiguate stimulus-processing from response-related P300 effects, given that response-evoked potentials are likely to contaminate the amplitude and/or latency of the P300. However, both stimulus- and response-driven effects would be led back to a priming effect. To our knowledge, this is the first evidence of a neural gender stereotype asymmetry for the automatic and implicit processing of faces. Thus, our findings corroborate previous evidence of gender stereotype asymmetry in language and further show it for the automatic processing of faces.

### Priming effects for target pronouns

Unfortunately, our results with pronouns were less conclusive. We only partially replicated our previous behavioural and ERP results on pronouns using the same material and paradigm (i.e. Siyanova-Chanturia *et al.*, 2012; Pesciarelli *et al.*, 2019). For the *grammatical* condition, we replicated a behavioural priming effect, but its neural correlate differed from the previously observed one. Instead of the P300 and N400 effects previously observed, an early effect consistent with the P300 effect (emerging from data-driven analyses) and a later positive component maximal at anterior sites and interpreted as the LPP showed sensitivity to grammatical gender violations (emerging from both *a-priori* and data-driven analyses). A larger positivity for grammatical gender agreement violations is in line with the definitional/grammatical effects found on the P600 (Osterhout *et al.*, 1997; Canal *et al.*, 2015; Su *et al.*, 2016) and it is interpreted considering that gender agreement violations between an anaphor and its antecedent could be processed like syntactic anomalies (Osterhout and Mobley, 1995). Here, the effects emerged only in response to the feminine pronoun (e.g. *inesperto*_masc_ ‘nonexpert’—*lei* ‘she’), limited to men for the LPP. These differ from the effect we previously observed, which was stronger for women and masculine pronouns (Pesciarelli *et al.*, 2019), and from the ones reported in literature, i.e. a larger P600 effect for gender agreement violations in women than men (Osterhout *et al.*, 1997) [but see Su *et al.* (2016)’s finding of a definitional incongruence effect on the P600 only for Chinese female reflexive pronouns]. A tentative explanation for the LPP effect could be that men were more sensitive than women to stereotypes related to their own gender, thus reacting to the violation of female pronouns. This sensitivity could have been somehow triggered by the presence of faces in the overall experiment, but, because it was unpredicted, it would need further investigation.

Instead, we failed to find effects, either behavioural or ERP, to *stereotypical* gender violations for pronouns. This null finding is reminiscent of that by Cacciari and Padovani (2007, exp.1). The RTs to gender-categorize the pronouns were in both studies unusually fast (mean ∼470 as compared with ∼550 ms in Pesciarelli et al. and Siyanova-Chanturia et al.) and both reported no stereotypical gender violation effects on the RTs. In a second study however, after instructing the participants to read and remember the primes for a later assessment, the RTs were longer and were affected by the stereotypical gender violation (exp.2). In Siyanova-Chanturia et al. and Pesciarelli et al., participants were instructed to ignore the prime or were unaware of its presence, but they started each trial by pressing the spacebar at the fixation cross. Here, participants were not given explicit instruction on the prime and each trial started automatically after 1000 ms from the previous. The absence of both manipulations could have produced an automatism in the processing of the target, weakening the effect of preceding stereotypical information. Alternatively, the intermixing of blocks with target pronouns and faces could have caused the stronger stereotypical effect for the face to inhibit the weaker effect for the pronoun, or a faster categorization of the pronouns which could have flattened any stereotypical effects. These possibilities remain speculative since this is the first study asking for a gender decision on both pronouns and faces in a stereotypical context. The absence of any behavioural or neural stereotypical effect for the pronoun precludes direct comparison with the face, but effects for pronouns have been largely replicated offering nevertheless a solid base for comparison.

## Conclusion

In conclusion, this study contributes to the understanding of the neural correlates of the implicit and automatic processing of faces in a stereotypical context and of the generality of the automatic gender-stereotyping effects independent of language. Considering that we typically *talk* about people that we *see*, and that stereotypes *automatically* activate in social interactions, our word-face priming paradigm is particularly suited to investigate stereotypes. Further, the human face is more relevant, ubiquitous, and rich in information than any other stimulus in our environment. Thus, examining gender stereotypes with faces using a priming paradigm allows us to better capture both its nature and its complexity. Note that the effects that we found are implicit and automatic to the extent to which the short presentation and response timing could not have allowed intentional or strategical processes to affect the behavioural or ERP responses. These effects could potentially differ from implicit and automatic effects found irrespective of the emphasis on gender or conscious awareness. However, our previous study using a masked and unmasked priming paradigm with pronouns (Pesciarelli *et al.*, 2019) suggests that they could be similar. Capitalizing on the multidimensionality of faces, this study offers a solid base for further investigation of the nature and functioning of the different biases.

## Supplementary Material

nsaf031_Supp

## Data Availability

Data will be made available upon request. Requests may be emailed to L.S. at luana.serafini@unimore.it or F.P. at francesca.pesciarelli@unimore.it.
