# Supplementary material for: The influence of gender stereotypical primes on the neural processing of words and faces
Source: Soc Cogn Affect Neurosci. 2025 Apr 16;20(1):nsaf031. doi: 10.1093/scan/nsaf031 (PMC12068222; doi:10.1093/scan/nsaf031)
Supplement: nsaf031_Supp [file nsaf031_supp.zip › scan-24-166-File011.docx]

# **Supplementary Material**

## **Supplementary methods**

### ***Participants***

Undergraduate students from the University of Modena and Reggio Emilia, Italy, and researchers’ acquaintances participated in the experiment. Of the total sample of forty-two participants, thirty-eight were right-handed, three were ambidextrous and one was left-handed, as assessed with the Edinburgh Handedness Inventory (Oldfield, 1971). Thirty-nine participants were native Italian monolingual speakers, two were native Italian bilingual speakers (with Polish and Arabic as other languages), and one was a native Romanian speaker with Italian as a second language (acquired from 6/7 years of age).

The experiment was approved by the local Ethical Committee (Comitato Etico dell’Area Vasta Emilia Nord- Italy) and it was run in accordance with the “Italian Association of Psychology” (AIP) Ethical Guidelines (Codice Etico: www.aipass.org/node/11560) and the Declaration of Helsinki.

### ***Stimuli***

Stereotypically female and male words received equally high evaluations of gender-oriented stereotypicality, whereas grammatically feminine and masculine words received low evaluations of gender-oriented stereotypicality (see details in Siyanova-Chanturia et al., 2012). Stereotypically male and female words and grammatically male and female words were matched for written frequency (La Repubblica corpus), length (number of characters), and rated valence.

Pronouns had a comparable written frequency. The two face exemplars were selected from the Chicago Face Database (Ma et al., 2015). The faces belonged to people of White racial background, posing frontally with neutral expression. Face stimuli were converted to greyscale, they were cropped to show the face, frontal hair, and ears, and they were assigned a black background. Face exemplars were selected from 206 faces rated for gender by an independent sample of 42 participants (21 female, age range = 19-31 yrs, M = 20.76 yrs, SD = 3.02 yrs). Participants rated on a 7-point-Likert scale how much each face was associated to a woman or a man (1 = woman, 7 = man). The scale was counterbalanced across participants. The selected faces were highly prototypical of the female (range = 1-4, M = 1.48, SD = 0.71) and male (range = 4-7, M = 6.62, SD = 0.66) gender categories, respectively. The same faces were also evaluated by the same sample for race reliability and attractiveness. Participants rated on a 7-point-Likert scale how much each face was associated with a White or a Black person (1 = White, 7 = Black), and how much each face was attractive (1 = not attractive at all, 7 = extremely attractive). The scales were counterbalanced across participants. The selected faces resulted highly prototypical of the White race category (female: range = 1-2, M = 1.24, SD = 0.43; male: range = 1-3, M = 1.40, SD = 0.59), and average on attractiveness (female: range = 1-7, M = 4.68, SD = 1.66; male: range = 1-7, M = 3.68, SD = 1.74).

### ***Design and procedure***

As in Pesciarelli et al. (2019), we presented all stimuli in contrast to a black background at the center of a 17” CRT monitor synchronous with the screen refresh [Philips 107B; refresh rate=60 Hz (16.67 ms)] that was located at eye level about 70 cm in front of the participant. Word stimuli (prime: 4-10 letter strings, target: 3 letter strings) were displayed in light grey uppercase letters (Couriel font, size 13) and they subtended 1.2–4.1° of horizontal visual angle and 0.5° of vertical visual angle. Face stimuli subtended 4.1° of visual angle. We used E-prime software (Version 3.0; Psychology Software Tools, Pittsburgh, PA) for stimulus presentation and behavioral response collection. The test computer was a HP Z2 Tower G5 Workstation with a CPU 2.80 GHz Intel® Core™ i9-10900 processor. Priority settings were optimized to ensure accurate display durations.

### ***EEG recording and analysis***

EEG was amplified and recorded with an ActiCHamp Plus (BrainProducts) system from 63 active Ag/AgCl electrodes (ActiCap Slim, BrainProducts) placed on the scalp according to the international 10–10 system. An additional three electrodes were placed around the eyes to control for eye movements (two were placed at the external ocular canthi and one below the left eye). EEG was digitized continuously with a sampling rate of 1000 Hz and a high cut-off of 280 Hz. FCz served as the recording reference and FPz as the ground.

Offline preprocessing was performed using BrainVision Analyzer software (Version 2.2.0; Brain Products, GmbH), following the procedure of Pesciarelli et al. (2019). EEG and EOG signals were downsampled to 500 Hz. EEG signal was re-referenced to the average activity of the two mastoids (TP9, TP10 electrodes) and band-pass filtered from 0.01 to 80 Hz using a zero-phase IIR Butterworth band-pass frequency filter (12dB/oct roll-off, 3dB cutoff frequencies, two-pass). Compromised channels were attributed the mean signal from 3 non-noisy nearby channels. A maximum of 1 channel was interpolated per participant (i.e., 1.59% of all channels). Eye-movement artifacts were corrected using a restricted infomax ICA (Bell & Sejnowski,1997), using a meaned slope detection algorithm (Gratton et al., 1983). Continuous EEG signal was segmented from -200 to 1000ms epochs time-locked to the target onset. The segmented signal was corrected against the 200ms pre-target baseline. Segments that contained artifacts (e.g., excessive muscle activity), as indexed by voltage steps > 75 µV, absolute voltage difference in 200ms > 200 µV, activity in 100ms < 0.5 µV, and absolute voltage > ±100 µV, were removed. We excluded from the analyses: two participants due to > 25% of overall rejected epochs, one participant due to > 50% of rejected epochs per condition, and three participants due to the high interference of alpha waves. Lost data due to artifacts of the remaining participants was equal to 5.04% (SD = 4.90%). Epochs associated with correct responses were averaged across the 16 conditions (mean number of epochs per condition = 27.81, SD = 2.76, range = 27.35- 28.21).

### ***Statistical analyses***

Statistical analyses were performed in JASP (Version 0.17.2.1; JASP Team, 2022). Behavioral analyses were conducted only on trials with response latencies within ± 2 SD from each individual mean, as in Siyanova-Chanturia et al. (2012) (rejected trials were on average 0.03%). ERP and behavioral analyses were conducted only on correct trials. Filler trials were excluded from the analyses. We found no significant correlation between the mean correct response times (RTs) and the proportion of correct responses in the face or pronoun condition (all *p*s >.10), suggesting the absence of a speed–accuracy trade-off (i.e., lower accuracy at faster RTs and higher accuracy at slower RTs). Thus, we separately analyzed accuracy and RTs.

For the planned comparisons, we used one-tailed paired-sample *t*-tests to assess the *a-priori* predictions that RTs were faster in the congruent than in the incongruent condition, and that mean ERP amplitudes were larger in the incongruent than congruent condition. For the N400 and LPP effects we tested separately the mean amplitude of the anterior and central clusters, while for the P300 effect we tested separately the mean amplitude of the central and posterior clusters. Clusters’ choice was based on the typical distribution of these effects (e.g., Šoškić et al., 2022) and on their distribution in previous studies using the same stimuli and paradigm (Pesciarelli et al., 2019; Siyanova-Chanturia et al., 2012). *P*-values are reported corrected for multiple comparisons with the Benjamini-Hochberg false discovery rate (FDR) control (Benjamini & Hochberg, 1995).

## **Implicit and explicit measures of stereotyping**

After the experiment, to measure individual stereotypical gender attitudes participants completed the *Bem Sex Role Inventory* (BSRI) (Gaudreau, 1977) and the *Ambivalence Sexism Inventory* (ASI) (Glick & Fiske, 1996). The BSRI has two different scales to measure: endorsement of desirable feminine personality characteristics and endorsement of desirable masculine personality characteristics. The ASI has two different scales to measure: sexist antipathy – *Hostile Sexism* (HS) and a subjectively positive orientation to women- *Benevolent Sexism* (HS). Those are two correlated components of sexism representing opposite evaluative orientations towards women.

To investigate the link between implicit and explicit measures of stereotyping we performed correlations of behavioral and ERP priming effects with individual BSRI and ASI subscales scores. Behavioral and ERP priming effects were calculated as the incongruent-minus-congruent RTs or mean ERP amplitude, respectively, in response to the male pronoun, female pronoun, male face, and female face separately, in the stereotypical condition. Mean ERPs were calculated at the anterior and central clusters for the N400 and LPP, and at the central and posterior clusters for the P300. The BSRI had missing values (in 6 participants: M_missing_ = 1.67 items, SD = 1.21 items, range = 1-4 items) due to negligence during completion. We replaced missing values at the item level with the mean value per subscale per participant, then calculated individual scores. We used Spearman’s correlation for non-normally distributed variables. *P*-values were corrected for multiple comparisons with the Benjamini-Hochberg FDR control (Benjamini & Hochberg, 1995).

No statistically significant correlation emerged between the ASI subscales (benevolent and hostile sexism) or the BSRI subscales (masculine and feminine) and the behavioral or the ERP priming effects for the stereotypical condition (all *p*s >.10). Thus, explicit gender stereotype beliefs seemed not to affect implicit gender stereotype processes, in line with a dissociation of explicit and implicit measures of stereotyping (Banaji & Greenwald, 1995; Banaji & Hardin, 1996).

# **References**

Banaji, M. R., & Hardin, C. D. (1996). Automatic stereotyping. *Psychological Science, 7*(3), 136–141. <https://doi.org/10.1111/j.1467-9280.1996.tb00346.x>

Bell, A. J., & Sejnowski, T. J. (1995). An information-maximization approach to blind separation and blind deconvolution. *Neural computation, 7*(6), 1129–1159. https://doi.org/10.1162/neco.1995.7.6.1129

Benjamini, Y., & Hochberg, Y. (1995). Controlling the False Discovery Rate: A Practical and Powerful Approach to Multiple Testing. *Journal of the Royal Statistical Society. Series B (Methodological), 57*(1), 289–300. <http://www.jstor.org/stable/2346101>

Gaudreau, P. (1977). Factor analysis of the Bem Sex-Role Inventory*. Journal of Consulting and Clinical Psychology, 45*(2), 299–302. https://doi.org/10.1037/0022-006X.45.2.299

Glick, P., & Fiske, S. T. (1996). The Ambivalent Sexism Inventory: Differentiating hostile and benevolent sexism. *Journal of Personality and Social Psychology, 70*(3), 491–512. https://doi.org/10.1037/0022-3514.70.3.491

Gratton, G., Coles, M. G., & Donchin, E. (1983). A new method for off-line removal of ocular artifact. *Electroencephalography and clinical neurophysiology, 55*(4), 468–484. <https://doi.org/10.1016/0013-4694(83)90135-9>

Greenwald, A. G., & Banaji, M. R. (1995). Implicit social cognition: Attitudes, self-esteem, and stereotypes. *Psychological Review, 102*(1), 4–27. <https://doi.org/10.1037/0033-295X.102.1.4>

Ma, D. S., Correll, J., & Wittenbrink, B. (2015). The Chicago face database: A free stimulus set of faces and norming data. *Behavior research methods, 47*(4), 1122–1135. https://doi.org/10.3758/s13428-014-0532-5

Oldfield R. C. (1971). The assessment and analysis of handedness: the Edinburgh inventory. *Neuropsychologia, 9*(1), 97–113. <https://doi.org/10.1016/0028-3932(71)90067-4>

Pesciarelli, F., Scorolli, C., & Cacciari, C. (2019). Neural correlates of the implicit processing of grammatical and stereotypical gender violations: A masked and unmasked priming study. *Biological psychology, 146*, 107714. <https://doi.org/10.1016/j.biopsycho.2019.06.002>

Psychology Software Tools, Inc. [E-Prime 3.0]. (2016). Retrieved from <https://support.pstnet.com/>.

Siyanova-Chanturia, A., Pesciarelli, F., & Cacciari, C. (2012). The electrophysiological underpinnings of processing gender stereotypes in language. *PloS one, 7*(12), e48712. <https://doi.org/10.1371/journal.pone.0048712>

Šoškić, A., Jovanović, V., Styles, S. J., Kappenman, E. S., & Ković, V. (2022). How to do Better N400 Studies: Reproducibility, Consistency and Adherence to Research Standards in the Existing Literature. *Neuropsychology review, 32*(3), 577–600. <https://doi.org/10.1007/s11065-021-09513-4>
